# Chemical Vapor Deposition Strategy of Fe‐N‐C Nanotubes for the Oxygen Evolution Reaction

**DOI:** 10.1002/advs.202413035

**Published:** 2025-04-15

**Authors:** Xin Liu, Tao Wei, Jonas Englhard, Maïssa Barr, Andreas Hirsch, Julien Bachmann

**Affiliations:** ^1^ Chemistry of Thin Film Materials Section Materials Chemistry Department of Chemistry and Pharmacy Friedrich‐Alexander‐Universität Erlangen‐Nürnberg (FAU) IZNF, Cauerstr. 3 91058 Erlangen Germany; ^2^ Department of Chemistry and Pharmacy & Joint Institute of Advanced Materials and Processes (ZMP) Friedrich‐Alexander University of Erlangen‐Nürnberg (FAU) Nikolaus‐Fiebiger‐Strasse 10 91058 Erlangen Germany

**Keywords:** atomic layer deposition, carbon‐based materials, chemical vapor deposition, electrocatalysis, nanotube, oxygen evolution reaction, precise chemistry

## Abstract

The conversion of metal‐nitrogen‐carbon (M‐N‐C) nanoparticles derived from conventional metal‐organic frameworks (MOFs) into self‐supporting and well‐defined metal‐nitrogen‐carbon (M‐N‐C) superstructures is essential for various functional applications but remains a significant challenge. In this study, a versatile chemical vapor deposition (CVD) strategy is developed for solvent‐free synthesis of self‐supporting carbonaceous nanotubes doped with metal and nitrogen (MNCT). The stable carbonaceous nanotubes doped with Fe and N (FeNCT) fabricated here exhibit excellent electrocatalytic performances for the oxygen evolution reaction (OER) and outperform the carbonaceous film doped with Fe and N grown on carbon foil directly (FeNC/CF), which demonstrates the advantages of the superstructure of FeNCT. This strategy also provides a way to tailor the metal‐nitrogen‐carbon nanotubes (MNCT) catalyst according to the feature of the reactor and exhibits many advantages, such as wide applicability and facile scalability.

## Graphical abstract

A CVD strategy is presented, an original preparative method, for generating heteroatom‐doped carbonaceous nanotubes via exclusively gas‐phase reactions. This strategy enables precise control of metal atom doping and geometric structures, enhancing electrochemical performance through synergistic effects. Optimized nanotubes achieve exceptional OER proficiency, above benchmark materials. This strategy serves as a versatile platform for advancing renewable energy storage and sustainable technology.

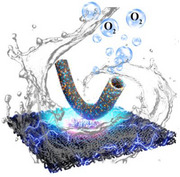

## 1 Introduction

Metal‐organic frameworks (MOFs) are a type of porous crystalline materials that have attracted widespread attention due to their diversity of metal nodes and ligands, high specific surface area, intriguing structure, and multifunctionality.^[^ ^1^, ^2^, ^3^, ^4^, ^5^, ^6^, ^7^, ^8^, ^9^, ^10^ ^]^ Benefiting from these advantages, MOF‐derived metal‐nitrogen‐carbon (M‐N‐C) composite materials have high porosity, large surface area, as well as uniformly doped active heteroatoms, making them widely applicable in energy conversion and storage, especially in electrocatalysis, such as the oxygen evolution reaction (OER),^[^ ^11^, ^12^, ^13^, ^14^, ^15^, ^16^, ^17^, ^18^, ^19^, ^20^, ^21^ ^]^ hydrogen evolution reaction (HER),^[^ ^22^, ^23^, ^24^, ^25^, ^26^, ^27^, ^28^, ^29^, ^30^, ^31^, ^32^ ^]^ oxygen reduction reaction (ORR),^[^ ^33^, ^34^, ^35^, ^36^, ^37^, ^38^, ^39^, ^40^, ^41^, ^42^, ^43^ ^]^ and other applications.^[^ ^44^, ^45^ ^]^ Currently, most reported M‐N‐C materials are obtained by direct pyrolysis of MOF crystal powders,^[^ ^46^, ^47^, ^48^, ^49^, ^50^, ^51^, ^52^, ^53^ ^]^ which is easy to operate, but is not optimal for electrocatalysis. First, MOF crystal particles are usually prepared by a solution‐based method,^[^ ^54^ ^]^ resulting in the generation of significant amounts of organic waste. Second, the introduction of active heteroatoms is mainly achieved either by the addition of metal precursors during MOF synthesis,^[^ ^11^, ^55^ ^]^ or based on the coordination of active heteroatoms with nitrogen‐carbon materials derived from MOFs.^[^ ^56^ ^]^ This makes it difficult to precisely control the amount of active metals. Third, MOF particles tend to aggregate at high pyrolysis temperatures, which is deleterious for the electronic conductivity of the resulting M‐N‐C material and for the transport of reactants and products during electrocatalytic reactions.^[^ ^57^ ^]^ Fourth, the subsequent electrode preparation processes involve coating the electrocatalyst onto a conductive substrate (such as carbon foil, glassy carbon electrode, etc.) with additional polymer binders, introducing an additional processing step. Furthermore, the presence of polymer binder agents not only partially covers active sites on the surface of the M‐N‐C, rendering them ineffective and catalytically inactive, but also hinders mass transport to the active sites. Finally, the limited mechanical stability and poor electrical contact quality of such pasted catalyst layers are usually drawbacks in HER and OER, as the generation of H_2_ or O_2_ bubbles causes the coated M‐N‐C to peel off the electrode. Therefore, developing a robust approach to creating M‐N‐C materials derived from MOFs with a superstructure and self‐supporting features toward OER remains a challenging but highly desirable task.

**Figure 1:**
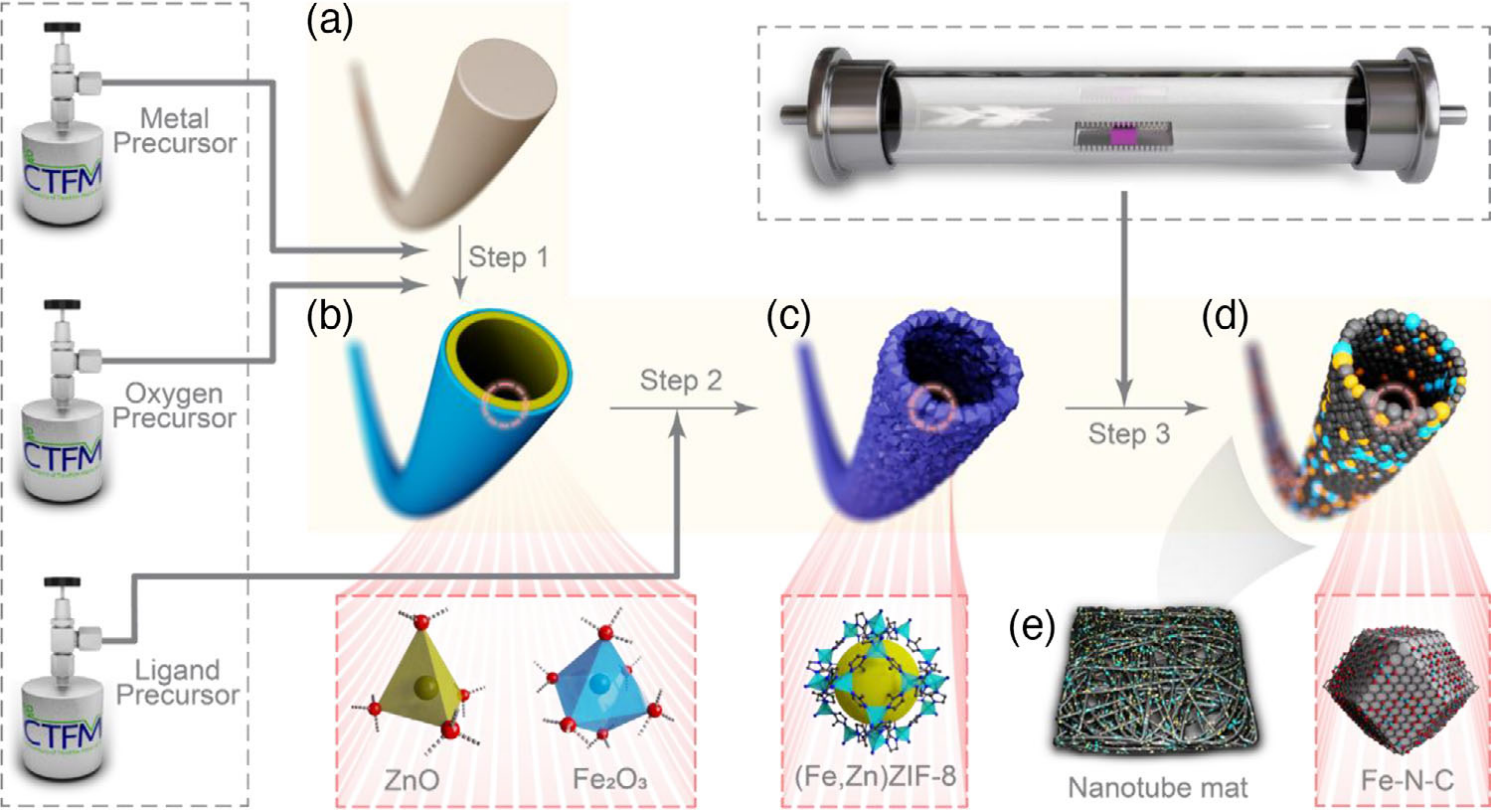
Schematic diagram of the synthesis of FeNCT by the new chemical vapor deposition strategy. Step 1: metal oxides are deposited on a polyacrylonitrile (PAN) nanowire (a, part of a mat obtained by electrospinning) to form oxide coatings by atomic layer deposition (ALD), using diethylzinc and water and *tert*‐butylferrocene and ozone as precursors. The first metal oxide layer is ZnO, followed by a layer of Fe_2_O_3_. Subsequently, PAN is removed (b) by exposure to ozone, O_3_, delivering Fe_2_O_3_/ZnO nanotubes. Step 2: Fe_2_O_3_/ZnO nanotubes (b) are converted to (Fe,Zn)ZIF‐8 nanotubes (c) in an extended gas‐induced process (GIP). The ligand precursor is 2‐methylimidazole (HmIM). Step 3: The (Fe,Zn)ZIF‐8 nanotubes are pyrolyzed to obtain Fe‐N‐C nanotubes (FeNCT) (d). The specific structures of nanotubes in b, c, and d are highlighted in the red boxes. (e) Free‐standing electrospun mat of FeNCT.

To address these challenges, we developed a chemical vapor deposition strategy (CVD strategy) for carbonaceous nanotubes doped with iron and nitrogen (**Figure** **1**). Earlier research demonstrated the conversion of ZnO to ZIF‐8,^[^ ^58^, ^59^, ^60^ ^]^ and the introduction of Fe into N‐C material to form Fe‐N‐C has also been investigated.^[^ ^61^ ^]^ To the best of our knowledge, the present study is the first to report the full process strategy of chemical vapor deposition (CVD) for synthesizing FeNCT from scratch. This CVD strategy consists of three steps: 1) atomic layer deposition (ALD) (Figure 1 Step 1), 2) subsequent gas‐induced process (GIP) (Figure 1 Step 2), and 3) a simple pyrolysis (Figure 1 Step 3). The ALD step yields self‐supporting Fe_2_O_3_/ZnO nanotubes. (Figure 1b) The subsequent gas‐induced process (GIP) transforms these Fe_2_O_3_/ZnO nanotubes into the self‐supporting iron‐doped ZIF‐8 nanotubes with a quantifiable and controllable amount of Fe doping, denoted as (Fe,Zn)ZIF‐8 nanotubes (Figure 1c). Finally, the pyrolysis process converts the (Fe,Zn)ZIF‐8 nanotubes into self‐supporting Fe‐N‐C nanotubes, named FeNCT (Figure 1d). The macrostructure of the FeNCT mat is a self‐supporting and hierarchical film consisting of nanotube‐shaped FeNCT (Figure 1e). The CVD strategy and as‐prepared FeNCT have several inherent advantages: 1) the ALD process allows for precise control of the growth of metal oxides and the doping with catalytically active transition metal ions; 2) the ALD process enables the growth of metal oxides on various templates with high aspect ratios, different shapes, and size, making it possible to tailor the catalyst for specific reactor shapes and characteristics; 3) With the inherent self‐supporting property and hierarchical structure, as‐prepared FeNCT can establish direct contact with conductive materials, ensure rapid electron transfer and excellent electrode structural stability, facilitate mass transport (electrolyte penetration and diffusion),^[^ ^62^, ^63^ ^]^ access active species easily, increase the electrochemically active surface area and accelerate the release of bubbles.

## 2 Results and Discussion

**Figure 2:**
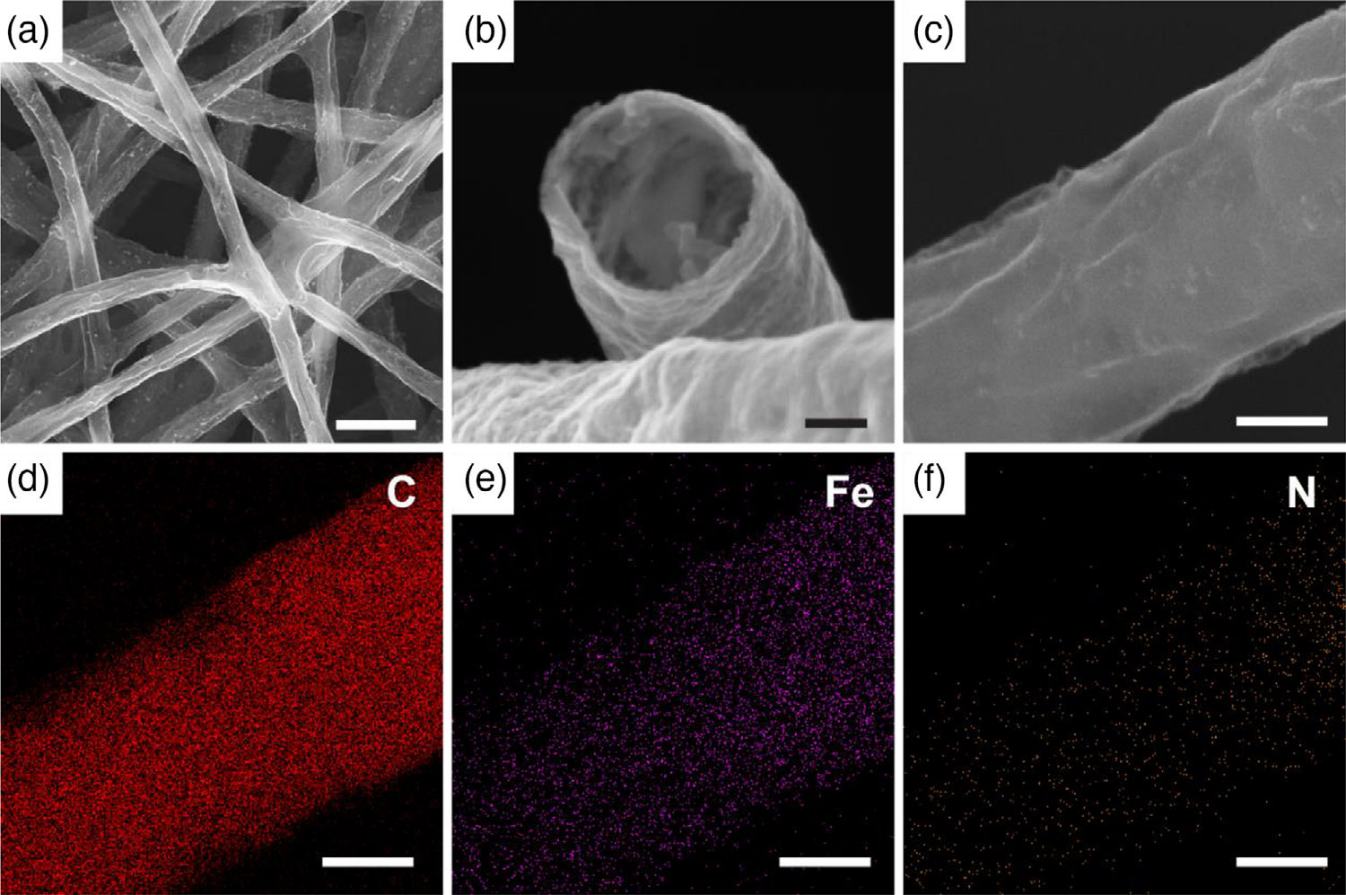
Characterization of FeNCT‐1000. a) Scanning electron microscope (SEM) image of FeNCT‐1000 electrospun mat. b) SEM image of FeNCT‐1000 cross‐section. c–f) SEM and EDS maps of FeNCT‐1000. The scale bar is 1 µm for a, 100 nm for b, and 200 nm for c–f.

Of the various metal oxide deposition protocols widely employed in nanofabrication, ALD was chosen to deposit metal oxide films on high aspect ratio features and control the thickness of deposited layers with precise accuracy at the nanoscale. Polyacrylonitrile (PAN) nanowires prepared by electrospinning from a PAN solution were chosen as a sacrificial template (Figure 1a; Figure S1a–d, Supporting Information). Due to its advantages of easy processing and removal, PAN nanowire mats can be produced on a large scale and removed completely under ozone and heating conditions. The PAN nanowires are placed inside an ALD reactor, and the precise growth of metal oxides on the surface of PAN nanowires is achieved due to the surface self‐saturation growth mechanism of ALD. The first layer is ZnO with 8 nm thickness, and the second layer is Fe_2_O_3_ with 1 nm thickness. Then the PAN nanowires were removed by prolonged O_3_ treatment in the ALD chamber at 200 °C to obtain Fe_2_O_3_/ZnO nanotubes (Figure 1b; Figure S1e–h, Supporting Information). Direct exposure of Fe_2_O_3_/ZnO nanotubes to 2‐methylimidazole (HmIM) vapor at 120 °C resulted in the generation of (Fe,Zn)ZIF‐8 nanotubes a highly uniform and fully covered tube wall (Figure 1c; Figure S1i–l, Supporting Information). These (Fe,Zn)ZIF‐8 nanotubes were then converted to FeNCT by simple pyrolysis at temperatures of 600, 800, and 1000 °C, resulting in the formation of FeNCT‐600, FeNCT‐800, and FeNCT‐1000, respectively. The FeNCT exhibits a morphology inherited from (Fe,Zn)ZIF‐8 nanotubes, characterized by a remarkable aspect ratio and thin walls (Figure 1d, **Figure** **2**a,b; Figure S1m–p, Supporting Information). Given our experimental parameters, the FeNCT are expected to be doped with Fe in the Fe/N molar ratio 0.03, a number that can potentially be adjusted via the number of ALD growth cycles performed (see Section S5 and Table S1, Supporting Information for the detail and full calculation of Fe doping).

As‐prepared FeNCT were investigated by energy‐dispersive X‐ray spectroscopy (EDS). The analysis of the elemental mapping of FeNCT‐1000 reveals that the distribution of the elements Fe, N, and C is uniform throughout the nanotubes, indicating uniform doping of Fe in the N‐C matrix (Figure 2c–f). The element Zn evaporates almost completely at these elevated temperatures. However, a small Zn signal remains in EDS and corresponds to remnants of either ZnO or coordinated Zn ions. Such Zn leftovers have been observed in similar cases in the past and are typically thought to be catalytically inactive.^[^ ^64^ ^]^ According to the data from EDS, the molar ratio of Fe/N is ≈0.048 (Figure S4 and Table S3, Supporting Information). Considering the uncertainty in EDS quantification, this ratio is consistent with the results calculated above.

**Figure 3:**
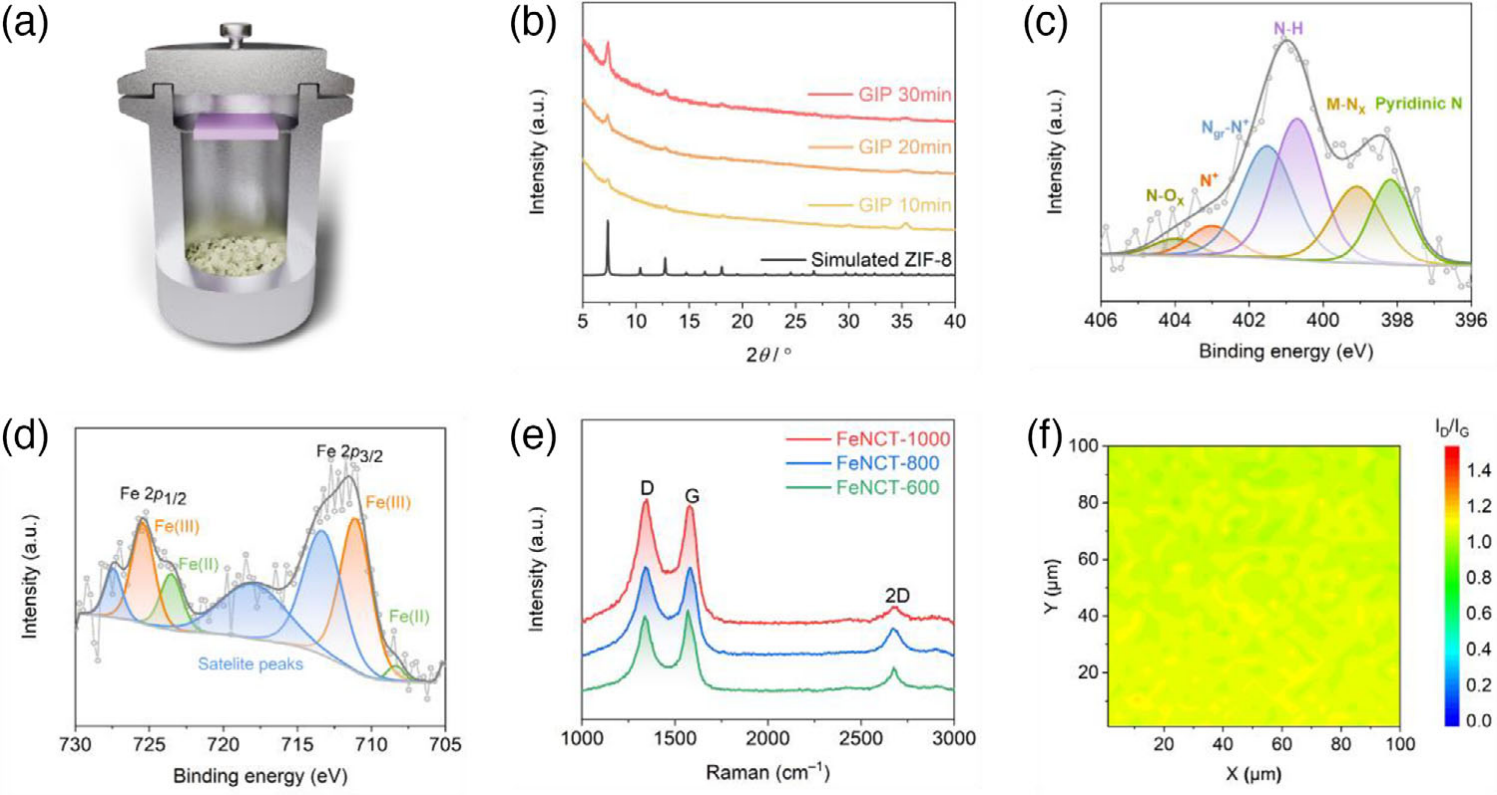
XRD, XPS, and Raman spectra. a) GIP reactor. b) X‐ray diffraction patterns of (Fe,Zn)ZIF‐8 nanotubes as a function of GIP reaction time and simulated pattern for ZIF‐8. c) XPS high‐resolution N1s spectrum with peak assignments of FeNCT‐1000. d) XPS high‐resolution Fe 2p spectrum with peak assignments of FeNCT‐1000. e) Raman spectra of FeNCT as a function of pyrolysis temperature. f) Raman mapping of FeNCT‐1000 mat I_D_/I_G_ ratio over a wide area of 100×100 µm^2^, using a step size of 2 µm (λ_exc_ = 532 nm).

The doping of Fe in ZIF‐8 was achieved during the transformation of Fe_2_O_3_/ZnO nanotubes into (Fe,Zn)ZIF‐8 nanotubes by the GIP step. This step is performed in a closed steel cylinder containing HmIM powder and preheated to 120 °C for 1 h in a drying oven then sealed in air with the sample (also preheated) and placed for the duration of the reaction in a drying oven (more detailed information is provided in the experimental section in the supporting information) (**Figure** **3**a). The key to achieving homogeneous doping is the thermal reaction of metal ions with 2‐methylimidazole (HmIM) vapor, during which they become mobile on the nanometer length scale,^[^ ^59^ ^]^ as well as the distinct reactivity of the two metal ions with HmIM vapor explored in our experiments. In the experiment, it was found that the reactivity of ZnO with HmIM vapor is higher compared to Fe_2_O_3_. This indicates that during the reaction, zinc ions preferentially coordinate with HmIM vapor to form an intermediate species, which eventually leads to the formation of ZIF‐8. Iron atoms are surrounded by a significant amount of these intermediates, providing favorable conditions for the doping of iron atoms in the coordination complex. To maximize the movement range of iron atoms upon the GIP step, a preliminary thermal mixing of Fe into ZnO was carried out, achieved by annealing the Fe_2_O_3_/ZnO nanotubes at 600 °C under Ar_2_ atmosphere. The X‐ray diffraction (XRD) patterns reveal the appearance of a diffraction peak corresponding to zinc ferrite (ZnFe_2_O_4_) after annealing, indicating the successful transport of Fe ions into the ZnO layer (Figure S6, Supporting Information). The degree of formation of (Fe,Zn)ZIF‐8 was found to be heavily reliant on the GIP reaction duration. To investigate this effect, three different reaction times were studied and denoted as “GIP *t*” (*t* = 10 min, 20 min, and 30 min). The XRD diffractograms reveal the advancement of the solid‐state reaction from the appearance of the first, rather broad ZIF‐8 peaks at 10 min (2*θ* values 7.34, 12.74, 18.06) along with a clear presence of unconverted ZnFe_2_O_4_ phase (2*θ* values 30.00, 35.67) via the intermediary appearance of the clearer ZIF‐8 phase with gradually disappeared ZnFe_2_O_4_ phase at 20 min to completion and exclusive presence of the ZIF‐8 phase after 30 min (Figure 3b).

The XPS N1s spectrum of FeNCT‐1000 reveals a characteristic peak at ≈399.5 eV (Figure 3c). This peak is commonly associated with the N‐metal interaction and provides evidence for the existence of Fe‐N_x_ bonds in the FeNCT‐1000 material. Based on the integration of the various peak components, the fraction of N atoms involved in Fe‐N_x_ bonding is ≈12%. Considering the Fe/N ratio of ≈0.03 mentioned above, it seems that essentially all the Fe present coordinates with N in a Fe‐N_4_ stoichiometry (Table S1, Supporting Information). XPS Fe 2p spectrum of FeNCT‐1000 reveals that Fe is present in oxidation states +II and +III, and can be fitted with 83% Fe(III) and 17% Fe(II) (Figure 3d). These N‐coordinated Fe centers are expected to provide catalytic activity. The high‐resolution N1s spectrum also reveals the existence of pyridinic N (≈398.1 eV) and graphitic N (≈401.5 eV).^[^ ^33^, ^61^, ^65^, ^66^ ^]^

To investigate the optimal temperature of pyrolysis on the OER electrochemical performance, three temperatures (600, 800, and 1000 °C) were studied. Raman spectroscopy exhibits temperature‐dependent I_D_/I_G_ ratio variations, going from 0.93 at 600 °C to 1.01 at 800 °C and 1.06 at 1000 °C (Figure 3e). The I_D_/I_G_ ratio is a widely used parameter to evaluate the degree of structural perfection in graphene‐based materials, where a higher value indicates a higher prevalence of defects.^[^ ^33^ ^]^ The observed increased trend in the I_D_/I_G_ ratio in the order of FeNCT‐600 < FeNCT‐800 < FeNCT‐1000 indicates the presence of abundant defects and graphitic carbon in FeNCT‐1000. These structural features can potentially facilitate the electrocatalytic process. The presence of these defects and graphitic carbon can be attributed to the high pyrolysis temperature used for the synthesis of FeNCT‐1000, which may have promoted the formation of these structural features in the material. These results provide valuable insights into the structure‐function relationships of FeNCT electrocatalysts. In addition, Raman *I*_D_/*I*_G_ mapping of the FeNCT‐1000 mat indicates a uniformly distributed *I*_D_/*I*_G_ ratio throughout the macroscopically sized mat (Figure 3f; Figure S7, Supporting Information).

**Figure 4:**
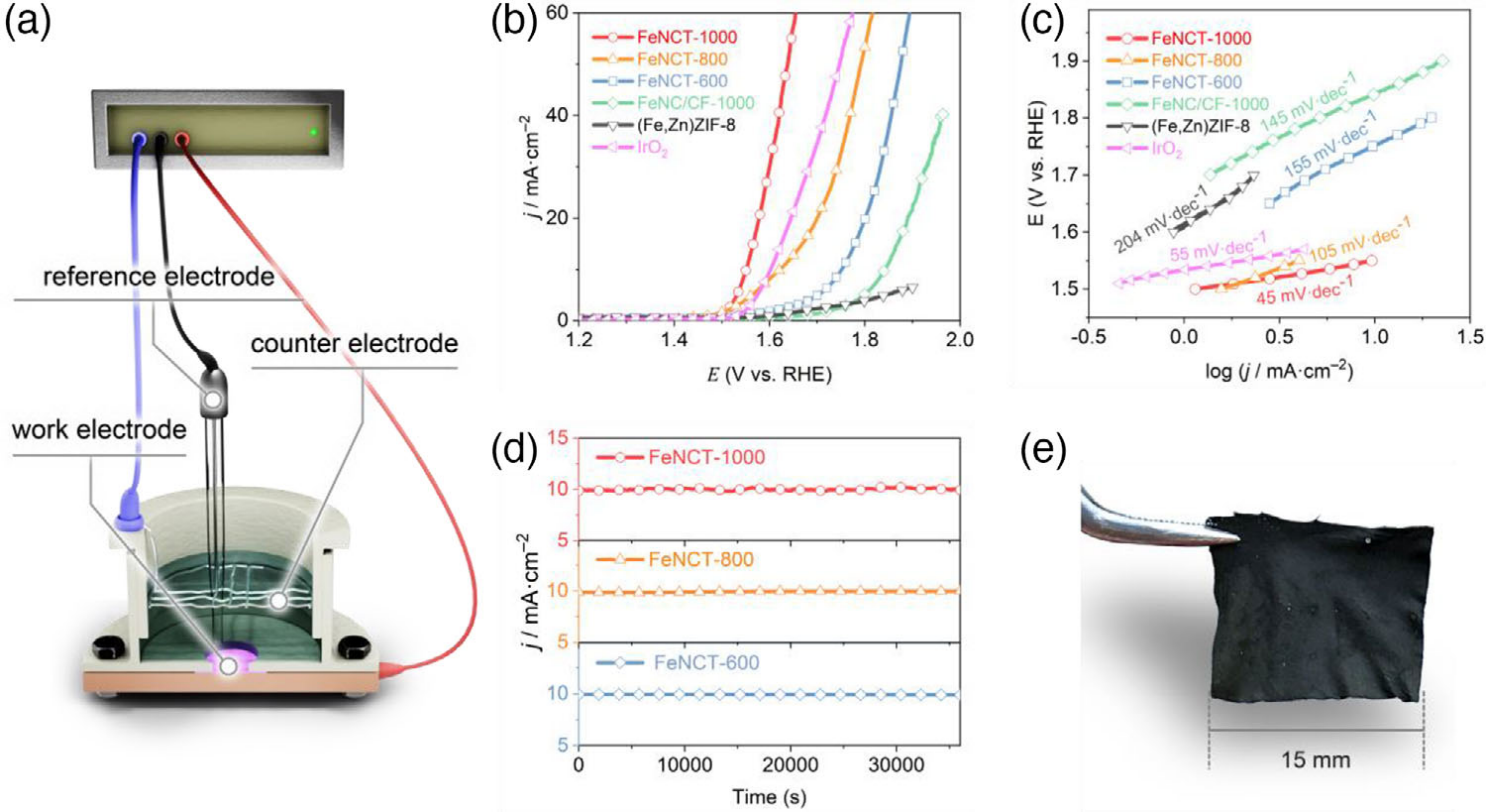
OER activity of FeNCT and its macroscopically sized mat. a) Home‐made electrochemical cell for OER activity testing. b) OER polarization curves of relevant electrocatalysts with a scan rate of 2 mV s^−1^. c) Tafel plot of relevant electrocatalysts highlighting the corresponding value of Tafel slopes with a scan rate of 2 mV s^−1^. d) Chronoamperometric tests for OER at a constant voltage of 1.55 V (FeNCT‐1000), 1.62 V (FeNCT‐800), and 1.75 V (FeNCT‐600) versus RHE. All the measurements are performed in 0.1 M KOH at room temperature, using a three‐electrode setup. e) Photograph of a sample of FeNCT‐1000 catalyst mat.

The electrocatalytic performance of FeNCT toward the oxygen evolution reaction (OER) was tested in an alkaline medium (0.1 M KOH) in a standard three‐electrode cell (**Figure** **4**a). Figure 4e shows the aspect of a typical FeNCT‐1000 sample of 15 mm × 15 mm size, tailored to our homemade electrochemical cell. Moreover, customization can be performed on various aspects, such as the shape, geometric dimensions, and thickness of the fibrous mat, as well as the diameter of the nanotubes. The thickness of the FeNCT‐1000 mat used here is ≈0.6 mm with a weight of ≈0.2 g cm^−2^. The loading of the mat with Fe is 2 × 10^−7^ g cm^−2^ (Table S2, Supporting Information). As a planar control, a carbonaceous film doped with iron and nitrogen on carbon foil was fabricated by our CVD strategy, resulting in the formation of a sample referred to as FeNC/CF‐1000 (Figure S5, Supporting Information).

The polarization curve of the FeNCT‐1000 mat shows that an overpotential of 320 mV (1.55 V versus RHE) is required to reach a current density of 10 mA cm^−2^ in 0.1 M KOH. This overpotential is lower than that of FeNCT‐800 mat, FeNCT‐600 mat, FeNC/CF‐1000, and even 5 mV lower than that needed with a commercial IrO_2_ catalyst (powder on carbon foil, the same weight as FeNCT mat, mixed with binder, Figure 4b, Figure S9 and Table S5, Supporting Information). The excellent catalytic activity of FeNCT‐1000 mat is also reflected in the Tafel slope value of 45 mV per decade in 0.1 M KOH. This value is lower than that of FeNCT‐800 mat, FeNCT‐600 mat, FeNC/CF‐1000, and even that of commercial IrO_2_ (Figure 4c; Figure S10 and Table S5, Supporting Information). The lower value compared to related MOF‐derived nanocarbon‐based electrocatalysts indicates its superior kinetics and correlates with its lower OER onset potential (see Table S6, Supporting Information for a comprehensive list of MOF‐derived nanocomposites and other relevant systems). Finally, FeNCT‐1000 demonstrates encouraging long‐term stability upon operation at 1.55 V, with a current density of 10 mA cm^−2^ maintained for at least 10 h at room temperature with no discernible signs of degradation as corroborated by the minimal difference of current density between the start and the end of the chronoamperometic test (Figure 4d; Table S4, Supporting Information). The morphology and internal structure of the sample are also maintained after this, as confirmed by SEM and Raman spectra (*I*_D_/*I*_G_ from 1.06 to 1.04, Figure S8, Supporting Information). These results show that the as‐prepared FeNCT‐1000 exhibits extraordinary activity for OER and durability.

FeNCT‐1000 exhibits an enhancement in electrochemically active surface area (ECSA) by 15% and 35% relative to FeNCT‐800 and FeNCT‐600, respectively (as determined from the voltammetry data based on the protocol described in the supporting information). Notably, the catalytic current densities measured at 1.55 V versus RHE revealed a more pronounced performance disparity, namely a current density of FeNCT‐1000 higher than FeNCT‐800 and FeNCT‐600 by 140% and 730%, respectively. The observed discrepancy between ECSA‐normalized enhancements and absolute current density improvements indicates that the enlarged ECSA of FeNCT‐1000 is insufficient to fully account for its substantial electrocatalytic activity enhancement (as reflected in current density). This implies that the primary origin of its enhanced electrocatalytic performance stems from intrinsic activity, which can be attributed to the synergistic effects of Fe‐N_4_ coordination chemistry and structural defects within the graphitized carbon matrix engineered through the CVD strategy. The presence of electrocatalytically active Fe centers within a predominantly sp^2^ nitrogen coordination environment (in Fe‐N_4_ stoichiometry) corresponds to what has been predicted by theoretical models to be the ideal structure.^[^ ^67^ ^]^

Furthermore, a statistically significant ECSA enhancement of 433% is observed for FeNCT‐1000 relative to FeNC/CF‐1000. Here, a high ECSA originates from its geometric superstructure of self‐supported nanotubes engineered through CVD strategy and observed by SEM. This superstructure integrates multiple advantageous features, including not only a high specific surface area but also enhanced accessibility to surface active species to the fluid phase, efficient electron transport along the conductive tubular framework, and inherent structural stability. An accelerated release of bubbles may be an additional advantage. This highlights the critical role of nanostructural designed by the CVD strategy in optimizing catalytic interfaces.

The trends observed conclusively demonstrate that the CVD strategy protocol influences the intrinsic catalytic activity, the ECSA, and the geometric superstructure comprising Fe‐N‐C nanotube and intertube spacing. These synergistic effects collectively contribute to the optimized oxygen evolution performance of the FeNCT‐1000 catalyst.

## 3 Conclusion

In summary, these results demonstrate the CVD strategy is a versatile strategy for metal‐ and nitrogen‐doped carbonaceous nanotubes (MNCT) and corresponding mats. This strategy 1) allows for quantitative control of the catalytically active metal dopant sites, 2) can tailor the MNCT morphology according to the feature of a reactor, 3) yields self‐supported MNCT with excellent electrical contact for direct application to OER, and 4) has the advantage of a solvent‐free preparation procedure. The FeNCT‐1000 mat can be directly used as an efficient OER electrode and exhibits higher activity, more favorable kinetics, and greater stability than traditional catalysts lacking the self‐supporting nanotube structure. The excellent electrocatalytic performance can be attributed to the synergistic effect of Fe‐N_4_ chemical composition, abundant defects within the graphitized matrix, high ECAS, and geometric superstructure, which increases the intrinsic activity, improves exposure and accessibility of active sites, enables better mass transfer and charge transport, and accelerates bubble release.

Several key aspects of our CVD strategy that directly impact the catalyst's performance and warrant further investigations in the future are highlighted:

1. Precise doping control via ALD (Atomic Layer Deposition): The use of ALD allows us to precisely control the thickness of the metal oxide layers, which in turn ensures the quantitative doping with active metal (Fe or further transition metals).
2. Impact of gas‐phase induced doping on Fe distribution: The reaction time during the gas‐phase induced process (GIP) is another crucial factor that controls the degree of Fe doping within the ZIF‐8 structure. Shorter reaction times result in incomplete mixing, while longer times ensure that Fe is fully incorporated into the ZIF‐8 framework. We expect that maximizing the density of active Fe‐N_4_ sites is key to the performance.
3. Defect engineering through carbonization temperature: The carbonization temperature is a key factor in the formation of defects within the graphitized carbon matrix. Higher carbonization temperatures (e.g., from 600 to 1000 °C) introduce more defects into the graphitized carbon framework. These defects, particularly in the form of vacancies and edge sites, serve as additional active sites that enhance the catalytic performance. As shown in our results, Fe‐N‐C nanotubes synthesized at 1000 °C exhibit more defects and higher OER activity than those pyrolyzed at lower temperatures.
4. Geometric superstructure engineered: The superstructure has been mechanistically validated as a critical determinant of enhanced electrochemical performance. The CVD strategy based exclusively on gas reaction, governed by a surface‐limited reaction mechanism, enables precise construction of template‐derived superstructure with inherited topological features. This synthetic paradigm achieved expanded ECSA, enhanced charge transport and mass transfer efficiency, and maximized accessibility to catalytically active sites through rationally designed spatial configurations.

Further development of this CVD strategy, inspired by recent advances in ALD/CVD technology, is expected to enable unmatched control over MNCT mat features, including a diversity of active sites and the potentially synergistic incorporation of multiple active metal centers and superstructure in the MNCT. Additionally, exploratory experiments suggest that the CVD strategy may be extended to MOFs composed of a wide range of metal nodes and ligands. The ALD step could potentially also be replaced by a (simpler) CVD coating of ZnO and Fe_2_O_3_, if the requirements in terms of thickness control and conformality are met. This broad applicability of our novel CVD strategy has the potential to accelerate the implementation of M‐N‐C materials in various applications, particularly in energy conversion and storage, marking a significant milestone in the development of devices for these fields.

## Conflict of Interest

The authors declare no conflict of interest.

## Author Contributions

X.L. performed conceptualization, investigation, formal analysis, data curation (primary contribution), and visualization, and wrote, reviewed, and edited the final manuscript. T.W. performed investigation, formal analysis, and data curation (Raman spectroscopy), wrote, reviewed, and edited the final manuscript. J.E., performed investigation (electrospinning), wrote, reviewed, and edited the final manuscript. M.B. performed investigation, formal analysis, and data curation (XPS). A.H. acquired resources. J.B. performed conceptualization, supervision, and resources, and wrote, reviewed, and edited the final manuscript.

## Supporting information



Supporting Information

## Data Availability

The data that support the findings of this study are openly available in Zenodo at https://zenodo.org/doi/10.5281/zenodo.13734293, reference number 13734293.
