# Supplementary material for: Chemical Vapor Deposition Strategy of Fe‐N‐C Nanotubes for the Oxygen Evolution Reaction
Source: Adv Sci (Weinh). 2025 Apr 15;12(20):2413035. doi: 10.1002/advs.202413035 (PMC12120779; doi:10.1002/advs.202413035)
Supplement: Supplementary file 1 — Supporting Information [file ADVS-12-2413035-s001.pdf]

ADVANCED SCIENCE
Open Access

Supporting Information

for *Adv. Sci.*, DOI 10.1002/advs.202413035

Chemical Vapor Deposition Strategy of Fe-N-C Nanotubes for the Oxygen Evolution Reaction

*Xin Liu, Tao Wei, Jonas Englhard, Maïssa Barr, Andreas Hirsch and Julien Bachmann**

Supporting Information

Chemical Vapor Deposition Strategy of Fe-N-C Nanotubes for the Oxygen Evolution Reaction

*Xin Liu, Tao Wei, Jonas Englhard, Maïssa Barr, Andreas Hirsch, Julien Bachmann**

Contents

S1. Materials 3
S2. Preparation of FeNCT (CVD Strategy) 3
S2.1 Electrospinning 3
S2.2 Step 1: Atomic Layer Deposition (ALD) 3
S2.3 Step 2: Gas-Induced Process (GIP) 4
S2.4 Step 3: Pyrolysis Process 4
S3. Preparation of FeNC/CF (CVD Strategy) 4
S3.1 Preheat carbon foil 4
S3.2 Step 1: Atomic Layer Deposition (ALD) 4
S3.3 Step 2: Gas-Induced Process (GIP) 5
S3.4 Step 3: Pyrolysis Process 5
S4. SEM and illustration of CVD strategy process. 6
S5. Quantified Doping of Active Metal Atom 7
S6. Spectroscopic Ellipsometry 10
S7. X-ray Photoelectron Spectroscopy (XPS) 11
S7.1 XPS overview spectrum 11
S7.2 XPS Fe 2p 11
S8. Scanning Electron Microscopy and Energy Dispersive X-ray Spectroscopy 12
S8.1 SEM-EDS of FeNCT-1000 12
S8.2 SEM image of FeNC/CF-1000. 13
S9. Grazing Incidence X-ray Diffraction (GI-XRD) 14
S9.1 X-ray diffraction pattern of Fe_2O_3/ZnO nanotubes after annealing 14
S10. Raman Spectroscopy 15
S11. Electrochemical Measurement 16
S11.1 OER polarization of FeNCT-1000 and commercial IrO_2. 17
S11.2 Tafel plot Tafel plot of FeNCT-1000 and commercial IrO_2. 17
S12. Electrocatalytic Performance of Selected as-prepared Electrocatalysts 19
S13. Comprehensive List of Literature Counterparts 20
S14. References 22

S1. Materials

Diethylzinc (DEZ) (≥95%), tert-butyl ferrocene (TBF), 2-methylimidazole, dimethylformamide (DMF) (99.8 %), iridium oxide powder (IrO_2), and polyacrylonitrile (PAN) with an average M_W of 150,000 were purchased from Sigma Aldrich Co. or abcr and used as received.

Silicon (100) wafers with an overlying oxide layer (approximately 200 nm) were purchased from Silicon Materials, Inc. They were then cut into the desired sizes before being cleaned with isopropanol in an ultrasonic bath before use.

S2. Preparation of FeNCT (CVD Strategy)

S2.1 Electrospinning

Preparation of PAN nanowire

The solution used for electrospinning was prepared by dissolving PAN (10 wt-%) in dimethylformamide. The dissolution process was accelerated by stirring the suspension at 80°C for 10 min. The solution was continuously stirred while cooling down to room temperature. Electrospinning was carried out in a Fluidnatek LE-10 instrument by Bioinicia, featuring a high-voltage power supply and a syringe pump. At ambient conditions, a positive potential of 20 kV was applied to the nozzle with a diameter of 22 ga, which was matched by a solution pump rate of 1.0 $mL\cdot h^{-1}$ to ensure a stable process.

The fibers were collected on aluminum foil wrapped around a grounded drum collector, which was spinning at 200 rpm and placed at a 15 cm distance to the nozzle. This drum rotation and a regular lateral movement of the spinning head ensured a homogeneous mat thickness all along the drum collector. Electrospinning was carried out for 1 h.

S2.2 Step 1: Atomic Layer Deposition (ALD)

Preparation of Fe_2O_3/ZnO nanotube

Commercial Gemstar-6 XT ALD reactor from Arradiance.

The ZnO thin films were deposited on PAN nanowire using DEZ and H_2O as ALD precursors. N_2 was used as both carrier and purge gas. The precursor DEZ and H_2O were at room temperature. The chamber temperature is 120 °C. The ALD cycle consisted of (1) pulse time of DEZ for 0.2 s, (2) exposure time of DEZ for 40 s, (3) purging with N_2 for 100 s, (4) pulse time of H_2O for 0.2 s, (5) exposure time of H_2O for 40s, (6) purging with N_2 for 100 s. The deposition Pressure is about 700 mTorr.

The Fe_2O_3 thin films were deposited on PAN nanowire using TBF and O_3 as ALD precursors. N_2 was used as both carrier and purge gas. The precursor TBF was evaporated at 65 °C and O_3

was at room temperature. The chamber temperature is 200 °C. The ALD cycle consisted of (1) pulse time of TBF for 2s × 4, (2) exposure time of TBF for 30 s × 4, (3) purging with N_2 for 100 s, (4) pulse time of O_3 for 0.5 s, (5) exposure time of O_3 for 60 s × 2, (6) purging with N_2 for 100 s. The deposition Pressure is about 700 mTorr.

Prolong the O_3 exposure time to 10 times more to remove the PAN completely at 200 °C.

S2.3 Step 2: Gas-Induced Process (GIP)

Preparation of (Fe,Zn)ZIF-8 nanotube.

Preheat Fe_2O_3/ZnO nanotube mat (1.5 cm × 1.5 cm) in a home-made steel cylinder reactor with 2-methylimidazole at 120 °C for 1h. Place the Fe_2O_3/ZnO nanotube mat and 2-methylimidazole in a home-made steel cylinder reactor, where Fe_2O_3/ZnO nanotube mat is on the top porous holder and 2-methylimidazole is at the bottom of the cylinder with the distance of 5 cm from the Fe_2O_3/ZnO nanotube mat to 2-methylimidazole. Then quickly close and seal the container and proceed with heating at 120 °C and for the durations stated in the main text. The GIP pressure in the cylinder is consistent with the saturated vapor pressure of 2-methylimidazole at 120 °C. Finally, remove the samples from the reactor and keep them in a 120 °C environment for another 2 h to remove residual 2-methylimidazole.

S2.4 Step 3: Pyrolysis Process

Preparation of FeNCT nanotube

The (Fe,Zn)ZIF-8 nanotube was used as a precursors to prepare the desired FeNCT catalyst via Pyrolyzed under Ar_2 atmosphere in a B180 tube furnace purchased from the company Nabertherm. The temperature was ramped up 5 °C·min^{-1} till 600 °C, 800 °C, 1000 °C and stayed in this temperature for 2 h, after which the sample was naturally cooled down to room temperature. As-prepared FeNCT is obtained.

S3. Preparation of FeNC/CF (CVD Strategy)

S3.1 Preheat carbon foil

Carbon foils were obtained from abcr GmbH and were subjected to a 2-hour thermal treatment at 1000 °C under Ar_2 flow before use, followed by natural cooling down to room temperature. This preheating step allowed for the dissipation of internal stresses in the foils, which is crucial for the homogeneous growth of catalytic layers.

S3.2 Step 1: Atomic Layer Deposition (ALD)

Preparation of Fe_2O_3/ZnO on carbon foil

Commercial Gemstar-6 XT ALD reactor from Arradiance.

The ZnO thin films were deposited on carbon foil using DEZ and H_2O as ALD precursors. N_2 was used as both carrier and purge gas. The precursor DEZ and H_2O were at room temperature. The chamber temperature is 120 °C. The ALD cycle consisted of (1) pulse time of DEZ for 0.2 s, (2) exposure time of DEZ for 40 s, (3) purging with N_2 for 100 s, (4) pulse time of H_2O for 0.2 s, (5) exposure time of H_2O for 40 s, (6) purging with N_2 for 100 s. The deposition Pressure is about 700 mTorr.

The Fe_2O_3 thin films were deposited on carbon foil using TBF and O_3 as ALD precursors. N_2 was used as both carrier and purge gas. The precursor TBF was evaporated at 65 °C and O_3 was at room temperature. The chamber temperature is 200 °C. The ALD cycle consisted of (1) pulse time of TBF for 2 s × 4, (2) exposure time of TBF for 30 s × 4, (3) purging with N_2 for 100 s, (4) pulse time of O_3 for 0.5 s, (5) exposure time of O_3 for 60 s × 2, (6) purging with N_2 for 100 s. The deposition Pressure is about 700 mTorr.

S3.3 Step 2: Gas-Induced Process (GIP)

Preparation of (Fe,Zn)ZIF-8 on carbon foil

Preheat Fe_2O_3/ZnO nanotube mat (1.5 cm × 1.5 cm) in a home-made steel cylinder reactor with 2-methylimidazole at 120 °C for 1h. Place the Fe_2O_3/ZnO nanotube mat and 2-methylimidazole in a home-made steel cylinder reactor, where Fe_2O_3/ZnO nanotube mat is on the top porous holder and 2-methylimidazole is at the bottom of the cylinder with the distance of 5 cm from the Fe_2O_3/ZnO nanotube mat to 2-methylimidazole. Then quickly close and seal the container and proceed with heating at 120 °C and for the durations stated in the main text. The GIP pressure in the cylinder is consistent with the saturated vapor pressure of 2-methylimidazole at 120 °C. Finally, remove the samples from the reactor and keep them in a 120 °C environment for another 2 h to remove residual 2-methylimidazole.

S3.4 Step 3: Pyrolysis Process

Preparation of FeNC/CF

The (Fe,Zn)ZIF-8 on carbon foil was used as precursors to prepare the desired FeNC/CF catalyst via Pyrolyzed under an Ar_2 atmosphere in a tube furnace purchased from company Nabertherm. The temperature was ramped up 5 $°C \cdot min^{-1}$ till 600 °C, 800 °C, 1000 °C and stayed in this temperature for 2 h, after which the sample was naturally cooled down to room temperature. As-prepared FeNC/CF-600, FeNC/CF-800, and FeNC/CF-1000 are obtained.

S4. SEM and illustration of CVD strategy process.

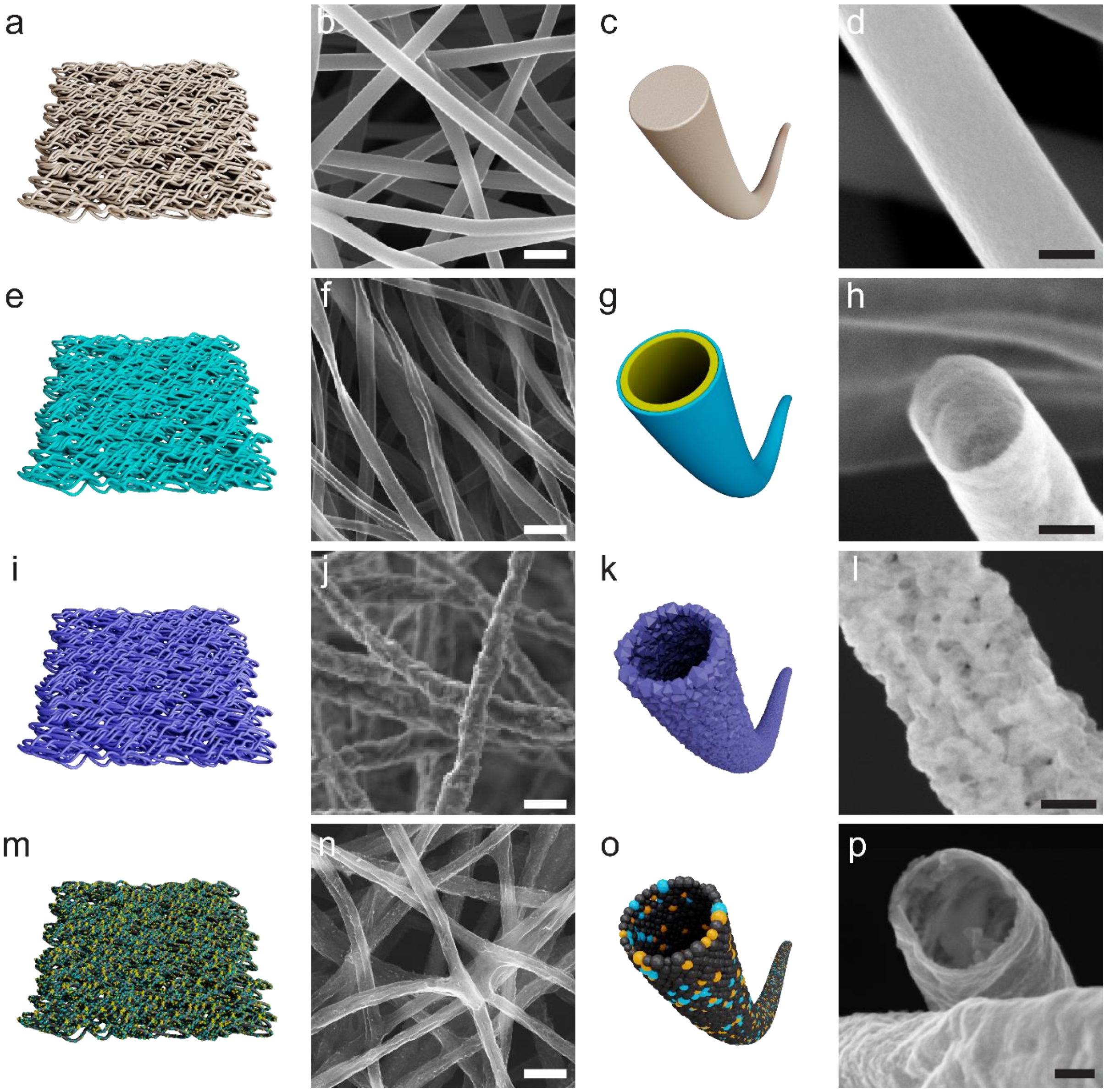

Figure S1 SEM and illustration of CVD strategy process. (a, b, c, and d) PAN nanowire; (e, f, g, and h) Fe_2O_3/ZnO nanotube; (I, j, k, and l) (Fe,Zn)ZIF-8 nanotube; (m, n, o, and p) FNCT. The scale bar is 1 μm for b, f, j, n, and 100 nm for d, h, l, p.

S5. Quantified Doping of Active Metal Atom

Assuming that the ALD film material grows as a void-free and crystalline film, the following equation can be used to calculate the quantity of material (in moles) deposited depending on reaction cycles in an atomic layer deposition (ALD) process:

$$n = K \cdot \frac{Z \cdot a}{V \cdot N_A} \cdot L \tag{1}$$

$$n = K \cdot \frac{Z \cdot a \cdot GPC}{V \cdot N_A} \cdot N \tag{2}$$

$$\frac{n_{(Fe_2O_3)}}{n_{(ZnO)}} = \frac{Z_{(Fe_2O_3)} \cdot V_{(ZnO)} \cdot GPC_{(Fe_2O_3)}}{Z_{(ZnO)} \cdot V_{(Fe_2O_3)} \cdot GPC_{(ZnO)}} \cdot \frac{N_{(Fe_2O_3)}}{N_{(ZnO)}} \tag{3}$$

$$\frac{n_{(Fe_2O_3)}}{n_{(ZnO)}} = 0.0725 \cdot \frac{N_{(Fe_2O_3)}}{N_{(ZnO)}} \tag{4}$$

$$\frac{n(Fe)}{n(Zn)} = 0.145 \cdot \frac{N(Fe_2O_3)}{N(ZnO)} \tag{5}$$

$$\frac{n(Fe)}{n(N)} = 0.03625 \cdot \frac{N(Fe_2O_3)}{N(ZnO)} \tag{6}$$

where:

L is the thickness loading of the material.

Z is the number of formula units per unit cell.

V is the unit cell volume.

N_A is Avogadro's constant.

GPC is the growth rate (increase in layer thickness per cycle).

N is Number of reaction cycles.

K is Unit conversion constant (converting to uniform units).

The parameters of the quantities mentioned in these equations can be found in **Table S1**.

Table S1 Crystallographic Data, Calculated Number of Reaction Cycles and Molar Ratio.

Quantity	Fe_2O_3 [1]		ZnO [2]
Z	6		2
$V\ [\mathring{A}^3]$	301.8		46.7
$GPC\,[nm \cdot cycle^{-1}]$	0.025		0.16
$N\,[cycle]$	40		50
$L\,(nm)$	1		8
$n(Fe){:}n(Zn)\big\|_{MO_x\ Film}$		0.12	
$n(Zn+Fe){:}n(N)\big\|_{(Fe,Zn)ZIF-8}$		1:4	
$n(Fe){:}n(N)\big\|_{(Fe,Zn)ZIF-8}$		~ 0.03	
$n(N_{(M-N_x)}){:}n(N)\big\|_{FeNCT-1000\ XPS}$		0.119	
$n(Fe){:}n(N_{(M-N_x)})_{FeNCT}$		~ 0.25	

Table S2 Calculated active metal density.

	Fe_2O_3
nm	1.00
cm^3/cm^2	1.00×10^{-7}
g/cm^3	5.24
g/cm^2	5.24×10^{-7}
g/mol	159.69

	Fe
g/mol	55.84
mol/cm^2	3.28×10^{-9}
Active metal density (g/cm^2)	1.83×10^{-7}

S6. Spectroscopic Ellipsometry

The thickness of the layer applied on a Si (100) wafer, used as a reference, was determined using a SENpro 39/030 spectroscopic ellipsometer from Sentech Instruments GmbH, along with SpectraRay 3 software. The ellipsometer collected data over a range of wavelengths from 370 nm to 1050 nm at an incident angle of 70°, and the resulting data was fit to a target model comprising the thickness and optical parameters of the film being analyzed.

S7. X-ray Photoelectron Spectroscopy (XPS)

X-ray photoelectron spectroscopy (XPS) was performed using an Al Kα source on a PHI Quantera II system. The resulting high-resolution spectra were processed by fitting individual peaks with the Shirley background and using Voigt functions. Since most of the carbon networks resembled graphene, the spectra were calibrated to C-C sp^2, which has a binding energy of 284.6 eV.

S7.1 XPS overview spectrum

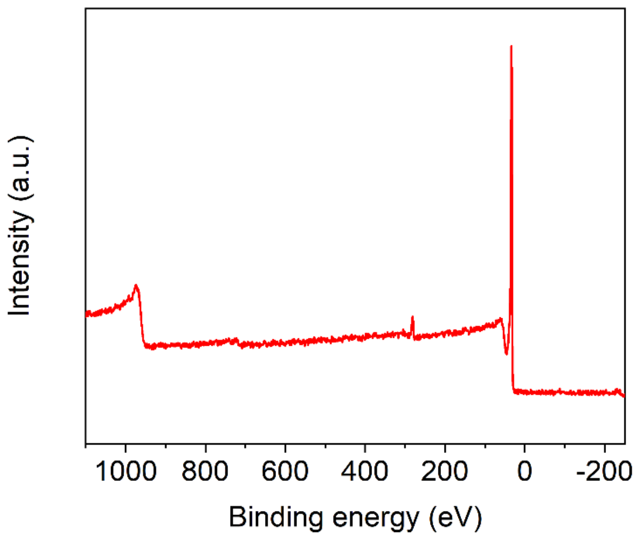

Figure S2. XPS overview spectrum of FeNCT-1000.

S7.2 XPS Fe 2p

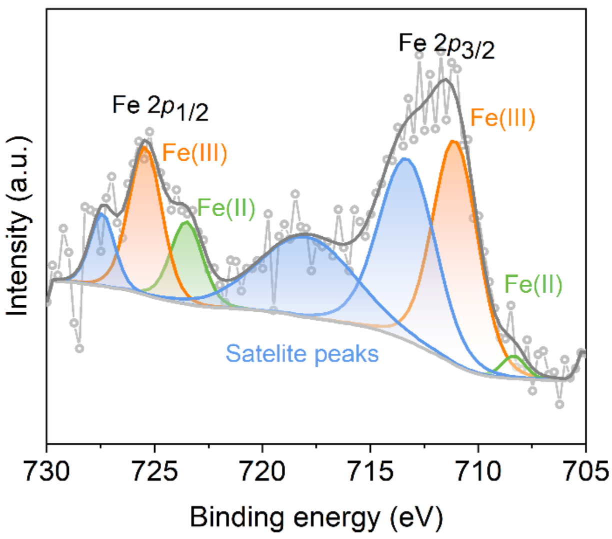

Figure S3. XPS high-resolution Fe 2p spectrum with peak assignments of FeNCT-1000.

S8. Scanning Electron Microscopy and Energy Dispersive X-ray Spectroscopy

Scanning electron microscopy was performed on AURIGA 40, manufactured by Carl Zeiss Microscopy GmbH, Oberkochen, Germany

Scanning electron microscopy energy dispersive X-ray spectroscopy was performed on a FE-SEM (Auriga, Carl Zeiss) equipped with an oxford X-max 80. The working conditions were set at an operating accelerating voltage of 15 KV, working distance of 6 mm, the elevation angle of the detector was 35° and the sample was vertically aligned with respect to the secondary-electron emission.

S8.1 SEM-EDS of FeNCT-1000

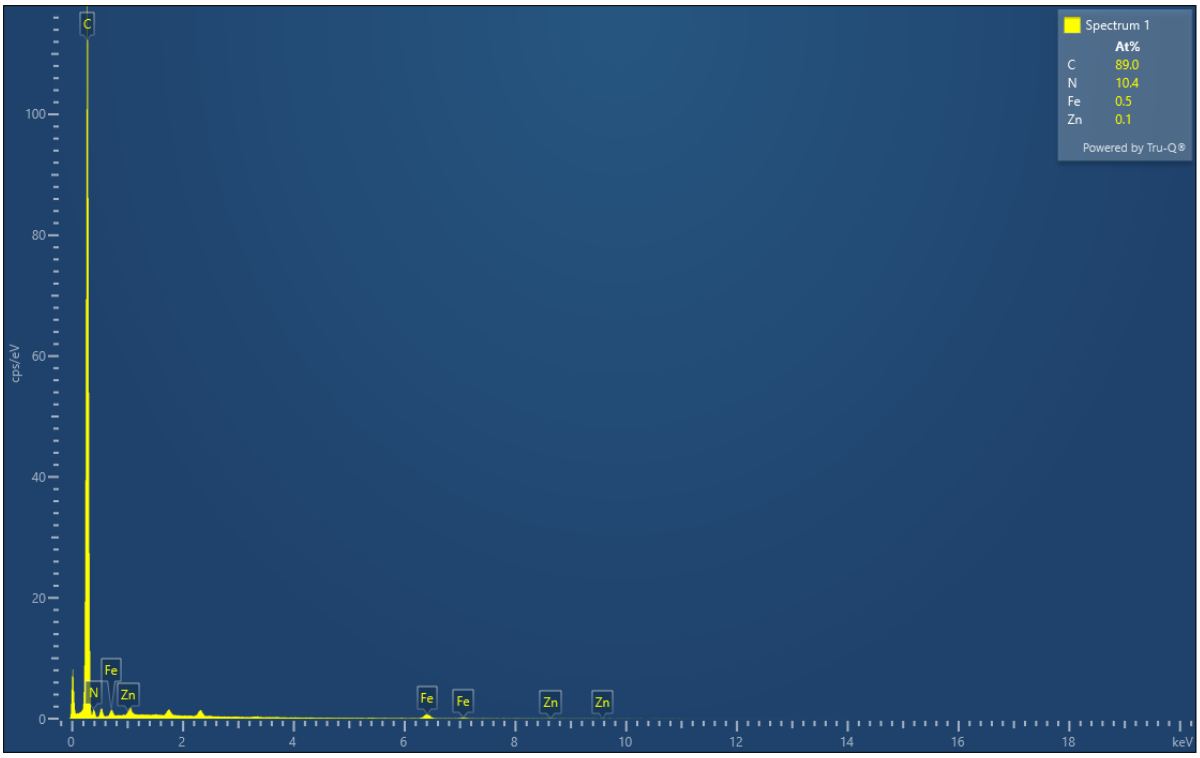

Figure S4. SEM-EDS of FeNCT-1000

Table S3. FeNCT-1000 SEM-EDS analysis.

Elements	Atomic %
C	88.95
N	10.44
Fe	0.50
Zn	0.11
Total	100

S8.2 SEM image of FeNC/CF-1000.

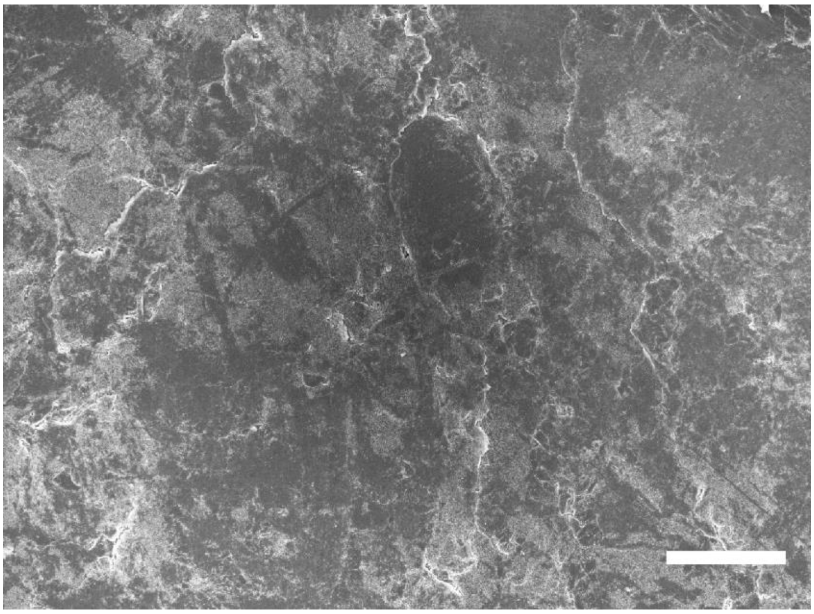

Figure S5 SEM image of FeNC/CF-1000. The scale bar is 200 μm.

S9. Grazing Incidence X-ray Diffraction (GI-XRD)

Grazing incidence X-ray diffraction (GI-XRD) was used to characterize the coatings of crystalline layers using a Bruker D8 Advance diffractometer. The radiation source used was Cu Kα, and the incident angle was set at 0.5°.

S9.1 X-ray diffraction pattern of Fe_2O_3/ZnO nanotubes after annealing

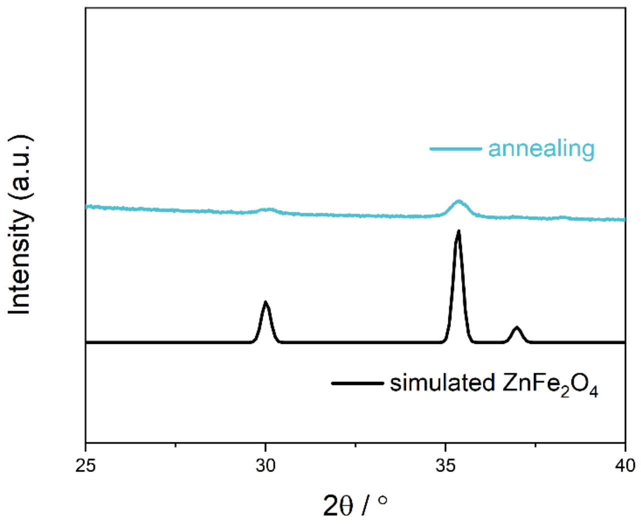

Figure S6 X-ray diffraction pattern of Fe_2O_3/ZnO nanotubes after annealing under N_2 at temperature of 600 °C for 15 min.

S10. Raman Spectroscopy

The confocal Raman microscope (WITec, alpha 300RA) equipped with a piezoelectrically-driven feedback-controlled scanning stage was used for statistical Raman spectroscopy measurements, resulting in a lateral positioning precision of 4 nm. The objective was employed to collect the backscattered light and elastically scattered photons (Rayleigh scattering) were obviated under the aid of a sharp-edge filter. Accurate sample movement control was offered by an automated xy-scanning table. Raman spectra were recorded with a lens-based spectrometer and a CCD camera (1024 × 128 pixels, cooled to -65 °C) utilizing a 600 lines·mm^{-1} grating. The diffraction limit focus yielded a lateral resolution of up to 200 nm.

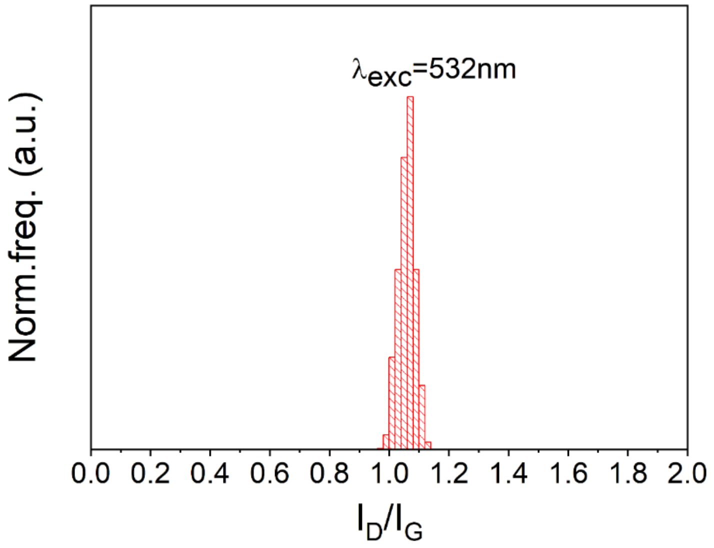

Figure S7 Averaged Raman spectra of FeNCT-1000 I_D/I_G ratio distribution histogram (λ_{exc} = 532 nm).

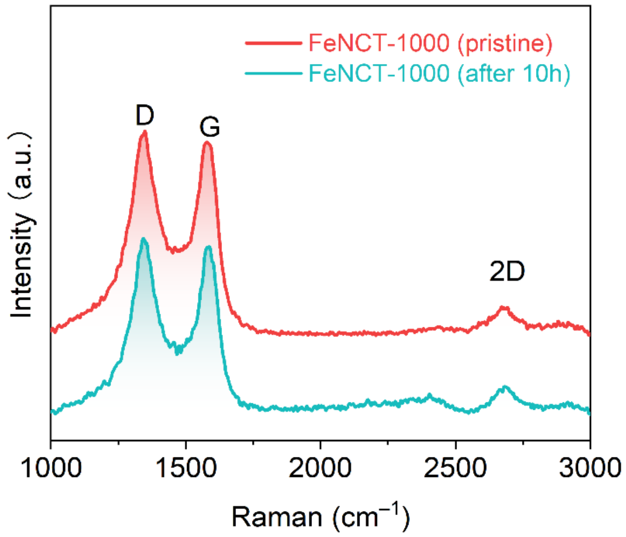

Figure S8 Raman spectra of FeNCT-1000 taken after 10 hours of electrolysis with a 1.04 I_D/I_G ratio.

S11. Electrochemical Measurements

The electrochemical evaluations for OER were carried out utilizing various techniques, including linear sweep voltammetry (LSV) and chronoamperometry using Gamry Interface 1000 potentiostats. The procedure began with 10 cycles of CV activation at a scan rate of 10 mV·s^{-1} between 1.0 V and 2.0 V vs. RHE, followed by LSV at a scan rate of 2 mV·s^{-1}. Some samples were subjected to test chronoamperometry at various applied potentials.

OER tests were performed using Gamry Interface 1000 potentiostats. All electrochemical measurements were conducted in a standard three-electrode system with Pt wires as the counter electrode, RHE or Ag/AgCl as the reference electrode, and FeNCT thin films as the working electrode. After several cyclic voltammetry (CV) tests to obtain stable curves, linear sweep voltammetry (LSV) for OER was performed at a scanning rate of 2 mV·s^{-1}. Stability investigations were conducted at a constant potential of 1.55 V vs. RHE in 0.1 M KOH. The current density was normalized to the geometrical area, and potentials were converted to versus RHE (reversible hydrogen electrode) according to the Nernst equation:

$$E_{RHE} = E_{Ag/AgCl} + 0.197V + 0.059PH$$

The Tafel slope was calculated using the following formula:

$$\eta = b\log(j/j_0)$$

Where:

η is the overpotential.

b is the Tafel slope.

j is the current density.

j_0 is the exchange current density.

S11.1 OER polarization of FeNCT-1000 and commercial IrO_2.

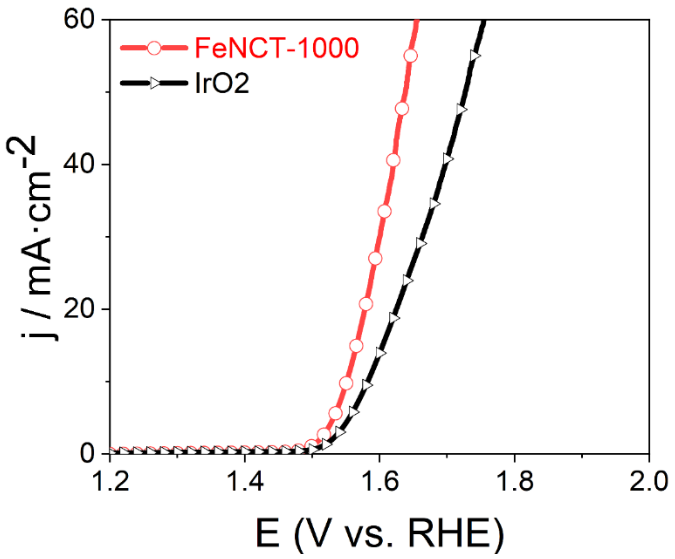

Figure S9 OER polarization comparison of FeNCT-1000 and commercial IrO_2. The onset potential of FeNCT-1000 is 1.55 V at 10 mA·cm^{-2}. After applying IR correction, the onset potential decreases to 1.54 V

S11.2 Tafel plot Tafel plot of FeNCT-1000 and commercial IrO_2.

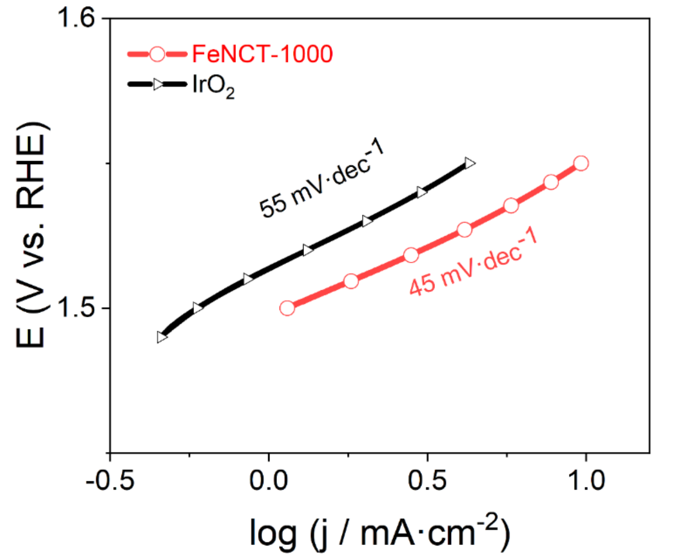

Figure S10 Tafel plot of relevant FeNCT-1000 and commercial IrO_2 electrocatalysts highlighting the corresponding value of Tafel slopes.

Table S4 the current density difference between the start and the end of the Chronoamperometric test for FeNCT-1000, FeNCT-800, and FeNCT-600.

Samples	Difference
	$mA\cdot cm^{-2}$
FeNCT-1000	0.07
FeNCT-800	0.11
FeNCT-600	0.06

S11.3 Electrochemically active surface.

To quantitatively assess the interfacial activity of the synthesized FeNCT, the electrochemically active surface areas (ECSAs) of FeNCT-1000, FeNCT-800, FeNCT-600, and FeNC/CF-1000 were systematically evaluated through electrochemical double-layer capacitance (C_{dl}) measurements. The C_{dl} values were derived from the average current (i) versus scan rate (v) in the non-faradaic potential region. Subsequently, the ECSA was calculated using the formula:

$$ECSA = \frac{C_{dl}}{C_s}$$

where C_s represents the specific capacitance per unit area. A standardized C_s value of 0.04 $mF\cdot cm^{-2}$ was adopted, consistent with literature protocols for analogous materials.

The ECSA values derived for FeNCT-1000, FeNCT-800, and FeNCT-600, and FeNC/CF-1000 were 1.04×10^4 $cm^2\cdot g^{-1}$, 9.03×10^3 $cm^2\cdot g^{-1}$, 7.67×10^3 $cm^2\cdot g^{-1}$, and 1.94×10^3 $cm^2\cdot g^{-1}$ respectively. FeNCT-1000 exhibits a 15% (1.15-fold), 35% (1.35-fold), and 433% (5.33-fold) enhancement in ECSA relative to FeNCT-800, FeNCT-600, and FeNC/CF-1000.

S12. Electrocatalytic Performance of Selected as-prepared Electrocatalysts

Table S5 Comparison of electrocatalytic performance of selected as-prepared electrocatalysts.

Composite	MOF Precursor	Medium	Potential@10mA·cm^{-2}	Tafel Slope
			V vs RHE	mV·Decade^{-1}
FeNCT-1000	(Fe,Zn)ZIF-8	0.1 M KOH	1.55	45
FeNCT-800	(Fe,Zn)ZIF-8	0.1 M KOH	1.62	105
FeNCT-600	(Fe,Zn)ZIF-8	0.1 M KOH	1.75	155
FeNC/CF-1000	(Fe,Zn)ZIF-8	0.1 M KOH	1.80	145

S13. Comprehensive List of Literature Counterparts

Table S6 comprehensive list of MOF-derived nanocomposites and other counterparts.

Composite	MOF Precursor	Medium	Potential@10mA·cm^{-2}	Tafel Slope	Reference
			V vs RHE	mV·Decade^{-1}	
FeNCT-1000	(Fe,Zn)ZIF-8	0.1 M KOH	1.55	45	This work
NC-MUV-3	Fe-ZIF-8 (MUV-3)	0.1 M KOH	1.561	48	Ref[3]
NC-ZIF-8	ZIF-8	0.1 M KOH	1.699	151	Ref[3]
NC-ZIF-67	ZIF-67	0.1 M KOH	1.626	71	Ref[3]
Fe-N_x/C	ZiF-8@Fe	1.0 M KOH	1.54	94	Ref[4]
Co-P/NC	ZIF-67	1.0 M KOH	1.549	52	Ref[5]
Fe-NC/rOCNT	Fe-ZIF/OCNT	0.1 M KOH	1.672	79.9	Ref[6]
S,N-Fe/N/C-CNT	/	0.1 M KOH	1.50	82	Ref[7]
CoFe-Co@PNC-12	ZIF-67@SiO2 @Fe	0.1 M KOH	1.55	81	Ref[8]
FeNC-S-Fe_xC/Fe	PANi CNFs-ZIF8-Zn	1.0 M KOH	1.553	/	Ref[9]
FeNC-Fe_xC/Fe	PANi CNFs-ZIF8-Zn	1.0 M KOH	1.606	/	Ref[9]
CoFe/N-GCT	/	0.1 M KOH	1.67	106	Ref[10]

Table S6 comprehensive list of MOF-derived nanocomposites and other counterparts.

Composite	MOF Precursor	Medium	Potential@10mA·cm^{-2}	Tafel Slope	Reference
			V vs RHE	mV·Decade^{-1}	
FeNi/N-CPCF-950	ZIF-8	0.1 M KOH	1.585	67	Ref[11]
N-GCNT/FeCo-3	/	0.1 M KOH	1.73	99.5	Ref[12]
Meso/micro-FeCoN$_x$-CN-30	/	1 M KOH	1.67	57	Ref[13]
FeNiCo@NC-P	Fe_2Ni MIL-88@ZIF ZnCo	0.1 M KOH	1.54	64	Ref[14]
CoZn-NC-700	Co,Zn ZIF-8	0.1 M KOH	1.62	69	Ref[15]

S14. References

[1] α-Fe2O3 hematite (Fe2O3 hem) Crystal Structure: Datasheet from "PAULING FILE Multinaries Edition – 2012" in SpringerMaterials (https://materials.springer.com/isp/crystallographic/docs/sd_1628103); Springer-Verlag Berlin Heidelberg & Material Phases Data System (MPDS), Switzerland & National Institute for Materials Science (NIMS), Japan: https://materials.springer.com/isp/crystallographic/docs/sd_1628103 (accessed.

[2] ZnO Crystal Structure: Datasheet from "PAULING FILE Multinaries Edition – 2012" in SpringerMaterials (https://materials.springer.com/isp/crystallographic/docs/sd_1714518); Springer-Verlag Berlin Heidelberg & Material Phases Data System (MPDS), Switzerland & National Institute for Materials Science (NIMS), Japan: https://materials.springer.com/isp/crystallographic/docs/sd_1714518 (accessed.

[3] J. López Cabrelles, J. Romero, G. Abellán, M. Giménez-Marqués, M. Palomino, S. Valencia, F. Rey, G. Mínguez Espallargas, *J. Am. Chem. Soc.* **2019**, *141*, 7173-7180.

[4] G. Li, J. Yang, Y. Chen, M. Liu, X. Guo, G. Chen, B. Chang, T. Wu, X. Wang, *ACS Appl. Mater. Interfaces* **2021**, *13*, 54032-54042.

[5] B. You, N. Jiang, M. Sheng, S. Gul, J. Yano, Y. Sun, *Chem. Mater.* **2015**, *27*, 7636-7642.

[6] J. Sheng, S. Zhu, G. Jia, X. Liu, Y. Li, *Nano Research* **2021**, *14*, 4541-4547.

[7] P. Chen, T. Zhou, L. Xing, K. Xu, Y. Tong, H. Xie, L. Zhang, W. Yan, W. Chu, C. Wu, Y. Xie, *Angew. Chem. Int. Ed.* **2017**, *56*, 610-614.

[8] Z. Lei, Y. Tan, Z. Zhang, W. Wu, N. Cheng, R. Chen, S. Mu, X. Sun, *Nano Research* **2021**, *14*, 868-878.

[9] Y. Qiao, P. Yuan, Y. Hu, J. Zhang, S. Mu, J. Zhou, H. Li, H. Xia, J. He, Q. Xu, *Adv. Mater.* **2018**, *30*, 1804504.

[10] X. Liu, L. Wang, P. Yu, C. Tian, F. Sun, J. Ma, W. Li, H. Fu, *Angew. Chem. Int. Ed.* **2018**, *57*, 16166-16170.

[11] Z. Wang, J. Ang, J. Liu, X. Y. D. Ma, J. Kong, Y. Zhang, T. Yan, X. Lu, *Applied Catalysis B: Environmental* **2020**, *263*, 118344.

[12] C. Y. Su, H. Cheng, W. Li, Z. Q. Liu, N. Li, Z. Hou, F. Q. Bai, H. X. Zhang, T. Y. Ma, *Advanced Energy Materials* **2017**, *7*, 1602420.

[13] S. Li, C. Cheng, X. Zhao, J. Schmidt, A. Thomas, *Angew. Chem. Int. Ed.* **2018**, *57*, 1856-1862.

[14] D. Ren, J. Ying, M. Xiao, Y. P. Deng, J. Ou, J. Zhu, G. Liu, Y. Pei, S. Li, A. M. Jauhar, H. Jin, S. Wang, D. Su, A. Yu, Z. Chen, *Adv. Funct. Mater.* **2020**, *30*, 1908167.

[15] B. Chen, X. He, F. Yin, H. Wang, D. J. Liu, R. Shi, J. Chen, H. Yin, *Adv. Funct. Mater.* **2017**, *27*, 1700795.
